# Psychosocial Factors Influencing Quality of Life After Spinal Cord Injury: A Scoping Review Between the United States and South Korea

**DOI:** 10.3390/healthcare14121736

**Published:** 2026-06-16

**Authors:** Hyun-Ju Ju, Debra A. Harley, Si-Yi Chao

**Affiliations:** 1 Counselor Education Program, Department of Counseling, Recreation and School Psychology, Florida International University, Miami, FL 33199, USA; 2 Department of Early Childhood, Special Education and Counselor Education, University of Kentucky, Lexington, KY 40506, USA; dharl00@uky.edu (D.A.H.); siyichao7185@uky.edu (S.-Y.C.)

**Keywords:** spinal cord injury, a cross-national review, quality of life, psychosocial adaptation

## Abstract

**Background**: Quality of life (QoL) after spinal cord injury (SCI) is influenced by psychosocial factors, yet less is known about how these factors are examined across national contexts. **Objective**: This scoping review mapped studies examining depression, employment, and social participation in relation to QoL or health-related QoL (HRQoL) among individuals with SCI in the United States and South Korea. **Methods**: Following PRISMA-ScR guidelines, five databases were searched for peer-reviewed English- and Korean-language studies published between 2007 and 2025. **Results**: Sixteen studies were included: nine from South Korea and seven from the United States. Depression and psychological distress were associated with lower QoL/HRQoL in both countries, although South Korean studies more often examined depression with stress and functional concerns, whereas U.S. studies situated depression within participation, spirituality, and youth psychosocial functioning. Employment was linked to QoL/HRQoL in both contexts, with South Korean studies emphasizing economic activity, vocational rehabilitation, and financial strain, and U.S. studies emphasizing employment status and vocational outcomes. Social participation was important in both countries, but South Korean studies focused more on community transition, functional independence, and social attitudes, whereas U.S. studies emphasized participation contexts, accessibility, and social relationships. **Conclusions**: Across the three domains, depression, employment, and social participation emerged as recurring psychosocial domains associated with QoL/HRQoL after SCI in both countries. These differences suggest that psychosocial adaptation after SCI should be understood within cultural and rehabilitation contexts.

## 1. Introduction

Spinal cord injury (SCI) is a term used to describe damage to the spinal cord resulting from various causes such as trauma (e.g., car accidents), diseases, or degeneration (e.g., cancer), or non-traumatic causes (e.g., toxins, tumors, infections) [1]. SCI is a life-altering disability that has a profound impact on an individual’s physical, psychological, and financial well-being [2]. That is, SCI can diminish the capacity to perform activities of daily living and self-care, although many restrictions in performing ADLs are also shaped by insufficient access to medical care, rehabilitation, and assistive technologies [1]. In terms of physical consequences, SCI leads to severe limitations in functionality [3,4]. Groah et al. [5] determined how SCI has affected the body’s system of composition in which standing, gait, muscle use, and not bearing weight all cause rapid alterations in the body’s composition that reduce lean muscle mass and increase fat. In addition, individuals with SCI commonly experience post-traumatic stress disorder (PTSD), depression, anxiety, self-stigma, public stigma, and ableism shortly after discharge from the hospital [6,7]. Furthermore, the treatment costs associated with SCI are notably high, encompassing expenses incurred during hospitalization and in rehabilitation centers [8].

The difficulties that people with SCI face in life are not limited to specific geographical regions but are prevalent worldwide. For example, in 2021, approximately 15.4 million people internationally were living with SCI and over 4.5 million living with long-term disability because of SCI. In addition, males are more commonly affected by SCI than females [1]. One of the negative social aspects is that more than 60% of adults with SCI experience unemployment globally [1]. The World Health Organization [1] also emphasized that saving lives, improving quality of life (QoL), and allowing people to be productive members of society are all important social and humanitarian goals. These perspectives collectively underscore the necessity of evaluating QoL as a crucial aspect in comprehending the enduring effects of SCI.

The epidemiological profile of SCI in South Korea directly shapes how QoL is ex-perienced by people with SCI and has shifted considerably over several decades. The mean age of individuals at the time of SCI rose from 32.3 years in 1985 to 52.8 years during 2012 to 2018, and falls have replaced traffic accidents as the leading cause of traumatic SCI, particularly among older adults [9]. Nationwide data confirm that the age-adjusted incidence of non-traumatic SCI increased from 24.11 per million in 2007 to 39.83 per million in 2020 [10]. These trends suggest that aging, falls, and secondary health conditions are increasingly important for understanding QoL among people with SCI in South Korea.

### 1.1. Quality of Life for Spinal Cord Injury

QoL encompasses various dimensions, including physical, psychological, and social aspects of an individual’s life [11]. However, QoL remains a complex construct in the SCI literature, and studies use a range of QoL and health-related quality of life (HRQoL) measures [12]. Therefore, the present review included studies examining either QoL or HRQoL to capture the multidimensional nature of long-term outcomes after SCI. As the life expectancy of people with SCI has increased significantly almost everywhere in the world, research has shifted toward long-term follow-up and consideration of QoL [13,14]. In line with this shift, factors impacting QoL have been comprehensively studied. For instance, previous studies have determined several key contributors, including level of injury, frequency of hospitalization, employment status, depression, spirituality, self-efficacy, optimism, hope, and physical functioning [12,15,16]. One area that substantially affects QoL for individuals with SCI is health literacy (HL), which varies not only from individual to individual but also culturally and globally. Individuals with excellent HL had significantly higher scores on the social and economic subdomains of QoL indicators compared to those with insufficient, problematic, or limited HL [17]. Given these impacts, QoL has been emphasized as a significant outcome measure for people with SCI. However, assessment measures are influenced by the tools used, the greater prevalence of quantitative rather than qualitative tools, a lack of a universal definition of QoL, and implicit or explicit reactions and evaluations of an individual’s life characteristics [12].

### 1.2. Theoretical Background: Psychosocial Adaptation to Disability

The psychosocial adaptation to disability has been considered as an important process in achieving QoL [18,19]. Livneh’s integrated psychosocial adaptation model explains how people with chronic illness and disability enhance QoL through antecedents, processes, and outcomes, while emphasizing that adaptation occurs across multiple functional and life domains, including affective, behavioral, domestic, and occupational domains [19]. Researchers have further emphasized that cultural and psychosocial factors may play a significant role in identifying cross-national differences in health context [20]. Furthermore, QoL is emphasized as a core health indicator that encompasses several areas, including psychological, social, and environmental domains [11]. Considering these points, reviewing cross-national contexts is helpful for identifying similarities and differences in psychosocial adaptation research, which remains less explored in the SCI literature.

### 1.3. Psychosocial Factors Affecting Spinal Cord Injury and Quality of Life in the United States and South Korea

In the United States, QoL is not simply an individual psychological outcome but is also a social structured environment. For instance, Whalley Hammell’s study [21] provides a significant conceptual foundation by demonstrating that QoL after SCI is defined by meaning, belonging, participation, and perceived autonomy. However, QoL factors and outcomes are not equally distributed across all populations that experience SCI. For example, it was reported that African Americans who have SCI showed poorer health outcomes as well as lower employment rates, with education and income as mediating variables [22]. Also, self-efficacy, social participation, acceptance of impairment, and depression were correlated and significantly predicted QoL in individuals with SCI [14,23]. In a qualitative study of factors influencing QoL in persons with long-term SCI, Roth et al. [24] examined how people attain and maintain optimal QoL over time. They found five themes: (1) injury, medical care, and rehabilitation, (2) built environment and accessibility, (3) relationships and support systems, (4) intrapersonal thoughts and emotions, and (5) handling challenges and adversity. The topics of importance evolved over time including from the injury and recovery process to navigating the community and handling difficult situations to self-reflection and handling challenges positively. Overall, U.S. research has examined psychosocial factors across a broad range of populations, including youth, adults, and veterans, with greater emphasis on sociodemographic diversity and participation. Conversely, in South Korea the emphasis and focus of QoL are perceived differently.

In South Korea, Korean participants prioritized bowel and bladder management and stress coping over sexual activity, unlike Western samples [25]. This suggests that functional and self-care are important aspects of QoL. Lee et al. [26] identified distinct QoL subgroups among community-dwelling South Koreans with SCI through latent profile analysis. They found that factors associated with a higher likelihood of belonging to the high-QoL group included marital status, fewer bowel dysfunction and muscle spasm or spasticity problems, receiving vocational rehabilitation services, currently engaging in paid work, less negative social attitudes, and better financial status. Employment status is also considered an important variable in QoL [27,28]. Some of these factors, such as employment and access to vocational rehabilitation, are similar to findings reported in the United States [29,30,31], suggesting that social integration may be a shared determinant of QoL across both countries. These findings also suggest that social participation is relevant in the South Korean context, as employment, vocational rehabilitation, environmental barriers, and negative social attitudes reflect the extent to which individuals with SCI can participate in community and social life. However, marital status, negative social attitudes, economic hardship, bowel dysfunction, and spasticity appear more prominently emphasized in the South Korean literature, suggesting that QoL in South Korea may also be shaped by family context, social perception, economic vulnerability, and health-management burden.

In a study predicting factors of QoL among persons with SCI in South Korea [32], QoL was associated with self-rated health, SCI duration, depressive symptoms, and fear of falling (FoF). Lee and Kim [33] conducted a cross-sectional survey of a Korean community SCI population and found that more health problems and lower QoL were more frequent among older individuals and patients without paid work. Together with prior Korean findings on bowel and bladder management, spasticity, and fear of falling, this suggests that secondary health conditions and aging-related health concerns are especially salient themes in South Korean SCI-related QoL research. Overall, these studies suggest some overlap between the U.S. and South Korean SCI literature, particularly in relation to psychological adjustment, employment, and social participation. However, these bodies of literature differ in emphasis. U.S. research has often highlighted autonomy, participation, sociodemographic disparities, and vocational rehabilitation outcomes, whereas South Korean research has more often emphasized depression, economic hardship, marital or family context, secondary health conditions, negative social attitudes, and community reintegration. These differences should be interpreted as differences in research emphasis and social context rather than direct evidence of national differences in QoL outcomes.

However, a systematic cross-national comparison of these psychosocial factors has not yet been conducted. The Korean SCI literature remains limited in scope and quantity, and the psychosocial constructs studied in South Korea differ substantially from those examined in the United States. It remains unclear which of these factors are shared across the two countries and which are specific to each national context.

Based on this literature review, the present study focuses on three psychosocial factors that commonly emerge in both U.S. and South Korean SCI research: depression, employment, and social participation. Although each country emphasizes different contextual issues, these factors provide a shared analytic framework for examining QoL/HRQoL after SCI. Together, they reflect psychological adjustment, socioeconomic role functioning, and community integration after SCI. Therefore, depression, employment, and social participation provide the analytic focus for the present scoping review.

The primary objective of this study is to map and synthesize how depression, employment, and social participation have been examined in relation to QoL/HRQoL among individuals with SCI in both the United States and South Korea. Additionally, this paper aims to explore the shared and distinct factors that contribute to the QoL of individuals with SCI in these two countries. The research questions addressed in the current study are as follows:

1. What is known about the relationship between depression and QoL/HRQoL among individuals with SCI in the United States and South Korea?
2. What is known about the relationship between employment and QoL/HRQoL among individuals with SCI in the United States and South Korea?
3. What is known about the relationship between social participation and QoL/HRQoL among individuals with SCI in the United States and South Korea?
4. What similarities and differences are evident in how these psychosocial domains are studied across the U.S. and South Korean SCI literature?

## 2. Method

### 2.1. Scoping Review and Search Strategy

A scoping review was selected because the research questions in this study align with the purpose of scoping reviews, which are suitable for addressing broader questions about what is known about the concept [34]. This scoping review was conducted and reported in accordance with the Preferred Reporting Items for Systematic Reviews and Meta-Analyses Extension for Scoping Reviews (PRISMA-ScR) guidelines [34]. No review protocol was developed or registered. For this review, five databases were used: PsycINFO, MEDLINE, CINAHL, the Korean Citation Index (KCI), and the Research Information Sharing Service (RISS). KCI and RISS were included to identify Korean-language or English-language publications from South Korea. Because international and Korean databases differ in language, scope, and indexing systems, search strategies were adapted accordingly. In PsycINFO, MEDLINE, and CINAHL, where U.S. and Korean studies in English are indexed, the search string was: (“spinal cord injury” OR “spinal cord injuries”) AND (“quality of life” OR QoL OR HRQoL OR “health-related quality of life”) AND (depression OR employment OR “social participation”) AND (“United States” OR USA OR U.S. OR Korea OR “South Korea”). Because Korean databases did not consistently support the full Boolean search string, separate domain-specific searches were conducted: (“spinal cord” AND “quality of life” AND depression), (“spinal cord” AND “quality of life” AND employment), and (“spinal cord” AND “quality of life” AND “social participation”). The publication period was limited to 2007–2025, aligning with the timeframe that reflects the epidemiological transition of SCI in South Korea. Recent analyses using national health insurance data demonstrated that the incidence of non-traumatic SCI (NTSCI) increased more rapidly than that of traumatic SCI (TSCI) between 2007 and 2020 [10], suggesting a substantial shift in the SCI population over this period [9]. Excel was utilized for the management of associated articles.

By focusing on QoL as the primary outcome, this study ensured theoretical coherence while still capturing the diverse psychosocial dimensions embedded in the literature. For the purpose of this review, the analysis focused on three psychosocial domains that appeared across both the U.S. and South Korean SCI literature: depression, employment, and social participation. Depression was treated as a psychological adjustment factor, employment as a vocational and socioeconomic factor, and social participation as a community and interpersonal participation factor.

### 2.2. Inclusion and Exclusion Criteria

Studies were included when they: (a) focused on people with spinal cord injury; (b) examined quality of life or health-related quality of life as an outcome; (c) examined depression, employment, or social participation in relation to QoL/HRQoL; (d) were conducted in the United States or South Korea; and (e) used quantitative, qualitative, or mixed-method designs. Studies were excluded when they were not SCI-focused, did not address QoL/HRQoL, did not examine one of the three psychosocial domains of interest, or were books, dissertations, review papers, editorials, or commentaries. Both English-language and Korean-language peer-reviewed articles were eligible for inclusion.

### 2.3. Study Selection, Data Charting, and Synthesis

**Figure 1:**
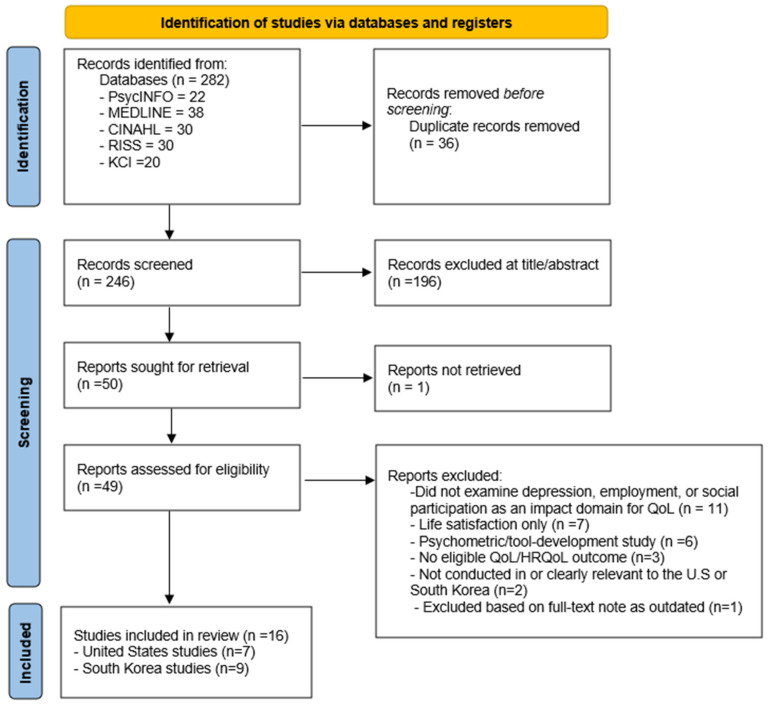
PRISMA flow chart.

The study selection process was documented using the PRISMA flow chart (see Figure 1). During full-text review, studies were assessed according to the inclusion and exclusion criteria described above. Formal critical appraisal was not conducted because this review aimed to map existing evidence.

Data was charted using a structured extraction form. Extracted information included author and year, country, study aim, population characteristics, age, duration of SCI when reported, study design, QoL/HRQoL measurement, psychosocial factors examined, and key findings. These data were summarized in Table 1 to allow comparison of study characteristics and psychosocial domains across the United States and South Korea.

The synthesis was conducted descriptively and thematically. First, studies were grouped by country. Second, findings were categorized according to the three focal psychosocial domains: depression, employment, and social participation. Third, shared and country-specific patterns were compared across the U.S. and South Korean literature. Because the included studies varied in design, sample characteristics, psychosocial constructs, and QoL/HRQoL measures, quantitative pooling was not conducted. Instead, the synthesis aimed to map how these psychosocial factors have been studied and to identify similarities, differences, and gaps across the two national contexts.

## 3. Results

A total of 282 records were identified through database searching. After removing 36 duplicates, 246 records remained. Of these, 196 were excluded based on title and abstract screening, and 49 full-text articles were assessed for eligibility. Subsequently, 36 articles were excluded according to the inclusion criteria, resulting in 16 studies being included in the final review (nine from South Korea and seven from the United States). See Table 1. The following is a summary comparing psychosocial factors on QoL of individuals with SCI between United States and South Korea.

### 3.1. Psychological Domain: Depression and Psychological Adjustment

South Korean studies in Table 1 most consistently highlighted depression as a key psychological correlate of QoL/HRQoL. Kim et al. [32] found that QoL was negatively correlated with depressive symptoms and SCI duration, and that fear of falling and SCI duration predicted QoL among SCI outpatients. Lee et al. [45] also reported that depression influenced QoL among young adults, while anxiety/depression and social factors influenced QoL among middle-aged adults. Other Korean studies linked broader psychological distress and interventions to QoL: Shin et al. [41] showed that HRQoL and employment status differed by psychological distress group among community-dwelling individuals with chronic SCI; Kim [42] reported that depression decreased and QoL improved after an efficient time-use intervention; Kim [43] found that QoL was lower among participants with stress and depression; and Kim and Kim [44] reported that cognitive behavioral therapy reduced depression and anxiety, increased self-efficacy, and improved QoL.

U.S. studies in Table 1 also linked depression and psychological adjustment to QoL, but the evidence was less concentrated on depression alone. Wilson et al. [36] found that spiritual well-being and depression were associated with QoL-related positive affect outcomes. Gorzkowski et al. [38] showed that broader social participation and job-related participation were associated with lower depression, which in turn was associated with higher QoL among girls with SCI. Thus, based on the included studies in Table 1, depression and psychological adjustment appeared in both countries, but South Korean studies more often examined depression alongside health-related or functional concerns, whereas U.S. studies more often connected psychological outcomes with participation, spirituality, and broader psychosocial adjustment.

### 3.2. Vocational Domain: Employment and Work-Related Participation

Employment and work-related participation appeared as important QoL-related factors in both South Korean and U.S. studies included in Table 1. In South Korea, Lee et al. [26] found that vocational rehabilitation services, paid work, less-negative social attitude, and better financial status were associated with high-QoL profile membership among community-dwelling individuals with SCI. Shin et al. [41] reported that employment status differed by psychological distress group, suggesting a link between work participation, HRQoL, and psychological adjustment. Kim [43] also found that QoL was higher among individuals with economic activity, and Kim [42] reported improved QoL alongside increased work and leisure time-use after intervention.

In the United States, employment was examined across adult, veteran, and vocational rehabilitation samples. Jain et al. [29] found that employment status was independently associated with HRQoL among adults with chronic SCI. Ottomanelli et al. [30] reported that assignment to supported employment did not significantly improve HRQoL, but obtaining competitive employment was associated with better social integration, mobility, and occupational outcomes among veterans with SCI. Chapin and Holbert [31] also found that employment at vocational rehabilitation closure was associated with higher overall QoL/health and higher physical, psychological, social relationship, and environmental QoL domains. Across Table 1, employment therefore functioned as both an economic factor and a psychosocial indicator of role participation, social integration, and adjustment after SCI.

### 3.3. Social Domain: Social Participation and Community Integration

Social participation and community integration were also evident across included studies, although they were studied somewhat differently across the two countries. In South Korea, social participation was often discussed through community transition, leisure, autonomy, disability acceptance, environmental barriers, and social attitudes. Moon et al. [39] reported that a hospital-based transitional rehabilitation program improved K-WHOQOL-BREF and functional independence, supporting the importance of community-transition-oriented QoL improvement. Han et al. [40] described leisure and social participation as central to QoL and well-being during COVID-19. Lee et al. [26] also identified less negative social attitude as part of the high-QoL profile, while Lee et al. [45] found that social factors such as income and religion influenced QoL among middle-aged adults with SCI.

In the United States, social participation was more often examined in relation to activity context, community living, accessibility, relationships, and social integration. Kelly et al. [37] found that participation context was more consistently related to QoL than participation frequency among youth with SCI, particularly for emotional, social, school, and overall psychosocial QoL. Bergmark et al. [35] showed that residence-related constraints and supports, including accessibility, finances, insurance, relationships, and personal assistance, shaped perceived QoL among adults with traumatic tetraplegia. Gorzkowski et al. [38] found that broader social participation was associated with lower depression and higher QoL, and Ottomanelli et al. [30] reported that competitive employment was associated with better social integration. Altogether, Table 1 suggests that social participation was a shared QoL-related domain, but U.S. studies more directly examined participation contexts and community-living conditions, whereas South Korean studies more often linked participation to community transition, social attitudes, and functional independence.

### 3.4. Cross-National Patterns Across Domains

Across the 16 included studies summarized in Table 1, depression, employment, and social participation provided a useful structure for comparing the U.S. and South Korean SCI-related QoL literature. Psychological factors appeared in both countries, but South Korean studies more frequently connected depression and psychological distress with stress, intervention, health-related burden, and other functional concerns [32,41,42,43,44,45]. U.S. studies connected depression and psychological well-being with participation, spirituality, and broader psychosocial outcomes [36,38]. Employment was also a shared domain, with South Korean studies emphasizing paid work, vocational rehabilitation, economic activity, and financial status [26,41,42,43], while U.S. studies emphasized employment status, competitive employment, and vocational rehabilitation outcomes [29,30,31]. Social participation appeared in both countries, but South Korean studies emphasized community transition, leisure, social attitudes, and functional independence [26,39,40,45], whereas U.S. studies more often examined participation context, residence, accessibility, relationships, and community living [35,37,38].

These findings indicate that the three domains were not isolated from one another. In several studies, psychological, vocational, and social factors overlapped. For example, Gorzkowski et al. [38] connected social and job-related participation with depression and QoL, while Shin et al. [41] linked psychological distress with both HRQoL and employment status. Similarly, Korean intervention and community-transition studies suggested that functional independence, work/leisure time-use, and social participation may affect QoL together [39,42]. Therefore, the domain structure should be interpreted as an analytic framework for organizing the evidence rather than as a set of mutually exclusive categories.

## 4. Discussion

This study identified and synthesized psychosocial factors associated with QoL/HRQoL among individuals with SCI in the United States and South Korea.

In the psychological domain, depression and psychological adjustment appeared in both South Korean and U.S. studies. In South Korea, Kim et al. [32] found that depressive symptoms were negatively associated with QoL and that fear of falling and SCI duration predicted QoL among SCI outpatients. Shin et al. [41] also showed that HRQoL and employment status differed according to psychological distress group among community-dwelling individuals with chronic SCI. Other Korean studies reported that depression decreased after time-use intervention [42], that stress and depression were associated with lower QoL [43], that cognitive behavioral therapy improved depression, anxiety, self-efficacy, and QoL [44], and that depression or anxiety/depression influenced QoL differently by age group [45]. In the United States, Wilson et al. [36] found that depression and spiritual well-being were associated with QoL-related positive affect, while Gorzkowski et al. [38] showed that broader social and job-related participation was associated with lower depression and higher QoL among girls with SCI. Taken together, the included studies suggest that psychological adjustment is a shared QoL-related domain, but South Korean studies more often focused directly on depression, stress, and health-related burden, whereas U.S. studies linked psychological outcomes with participation, spirituality, and youth psychosocial functioning. Therefore, the psychological domain highlights the importance of assessing and treating depressive symptoms as part of a broader process of adapting to injury, functional change, and long-term disability.

In the vocational domain, employment and work-related participation were also important in both countries. In South Korea, Lee et al. [26] found that paid work, vocational rehabilitation services, better financial status, and less negative social attitude were associated with higher-QoL profiles among community-dwelling adults with SCI. Shin et al. [41] linked employment status with psychological distress and HRQoL, and Kim [43] reported higher QoL among individuals with economic activity. In the United States, Jain et al. [29] found that employment status was independently associated with HRQoL among adults with chronic SCI. Ottomanelli et al. [30] reported that obtaining competitive employment was associated with better social integration, mobility, and occupational outcomes among veterans with SCI. Chapin and Holbert [31] also found that employment at vocational rehabilitation closure was associated with better overall QoL and better physical, psychological, social, and environmental QoL domains. These findings support employment as more than an economic variable. Across the included studies, employment was connected to social role participation, independence, integration, and psychological adjustment. This finding aligns with prior SCI research suggesting that employment and vocational rehabilitation are closely related to reintegration and long-term adjustment after SCI [27,28]. In this sense, employment should be understood not only as a marker of financial stability, but also as a psychosocial indicator of restored social role, autonomy, and participation in community life.

In the social domain, social participation and community integration were studied in both South Korea and the United States, although the emphasis differed by country. In South Korea, Moon et al. [39] showed that a hospital-based transitional rehabilitation program improved K-WHOQOL-BREF scores and functional independence, supporting the role of community-transition-oriented rehabilitation. Han et al. [40] described leisure, autonomy, disability acceptance, and social participation as central to QoL and well-being during COVID-19. Lee et al. [26] further showed that negative social attitude and financial status were associated with QoL profiles, and Lee et al. [45] suggested that social factors such as income and religion influenced QoL in middle-aged adults with SCI. In the United States, Kelly et al. [37] found that participation context was more consistently related to youth QoL than participation frequency. Bergmark et al. [35] showed that residence constraints and supports, including accessibility, finances, insurance, relationships, and personal assistance, shaped perceived QoL among adults with traumatic tetraplegia. Gorzkowski et al. [38] also connected social participation with lower depression and higher QoL. These included studies suggest that social participation is a core QoL-related domain in both countries, but U.S. studies more often examined participation through youth, veteran, and community-living contexts, whereas South Korean studies more often connected social participation with rehabilitation transition, leisure, negative social attitudes, and community reintegration.

These findings are consistent with the broader SCI literature suggesting that community participation, accessibility, environmental support, and social relationships are important determinants of QoL after SCI [46,47,48]. Thus, social participation should not be understood only as individual participation behavior, but also as a reflection of broader environmental and social opportunities that facilitate or restrict participation after SCI.

Across the three domains, the included studies suggest both shared and country-specific patterns. Depression, employment, and social participation were recurring domains in both the U.S. and South Korean SCI literature. However, the included South Korean studies more often connected QoL with depression, stress, fear of falling, functional independence, economic activity, vocational rehabilitation, and social attitudes. The included U.S. studies more often examined employment, participation context, community living, social and job-related participation, and veteran or youth populations. These patterns should be interpreted as differences in research emphasis and available evidence rather than direct evidence that people with SCI in the two countries experience QoL differently.

### 4.1. The Limitations of This Study

First, there were differences in the amount, scope, and type of literature from South Korea and the United States. Although RISS and KCI were used as additional Korean databases and Korean-language publications were included, differences in publication language, indexing systems, and database coverage may still have influenced which studies were identified. Therefore, the findings may not fully represent all SCI-related QoL research in either country.

Second, intracultural differences were not addressed thoroughly through this review. Such within-country variations may influence how individuals with SCI perceive their condition and adjust to life after injury. For instance, living in a location (e.g., urban or rural) can account for the difference among people with SCI. So, future research can explore adding more intracultural factors for cross-cultural review.

Third, our study did not conduct a consultation exercise as Levac et al. [49] suggested for the scoping review process last step. This may also provide valuable perspectives, meaning, and applicability to a study’s findings [50]. However, Arksey and O’Malley [51] listed consultation as highly encouraged but optional, and this study is a preliminary review for initially understanding factors. The use of a consultation exercise could improve this study because practical voices can be determined.

Fourth, the lack of quantitative comparative analysis between the United States and South Korea is a notable methodological limitation. Since this study employed a scoping review methodology, the primary goal was to map and summarize the existing literature rather than to quantify specific relationships between variables [52,53]. The 16 included studies differed substantially in study design, populations, psychosocial domains, and QoL/HRQoL measurement tools. For example, the South Korean studies included latent profile analysis, intervention studies, qualitative interviews, secondary analysis, and age-group comparison [26,32,39,40,41,42,43,44,45], whereas the U.S. studies included cohort, qualitative, survey, structural equation modeling, and vocational rehabilitation studies [29,30,31,35,36,37,38]. Because these studies examined different populations and outcomes, direct quantitative synthesis across the two countries was not methodologically appropriate. Future studies should consider using comparable QoL/HRQoL instruments and consistent psychosocial measures across countries to enable more systematic quantitative comparison of how psychosocial factors influence QoL among people with SCI.

Fifth, this review did not fully address the potential confounding factors that may have influenced the psychosocial factors and QoL outcomes identified in the reviewed studies. For instance, injury level and completeness, time since injury, age, gender, socioeconomic status, and access to rehabilitation services are all factors known to affect psychosocial outcomes in people with SCI [12,15]. The reviewed studies varied considerably in how they reported and controlled for these variables. This variation makes it difficult to determine whether the identified psychosocial factors are the primary influences on QoL or whether background variables also played a significant role. Therefore, future research should consider more consistent reporting of participant characteristics and control for potential confounding variables to better understand the independent effects of psychosocial factors on QoL across national settings.

### 4.2. Implications of This Study

The results of this review have several implications. The three-domain structure of psychological, vocational, and social factors may help researchers and clinicians organize the main areas that shape QoL/HRQoL after SCI. Since sociocultural is one of the antecedents of a psychosocial adaptation process for achieving a good QoL [19], a cross-cultural approach is required for understanding psychosocial adaptation.

For rehabilitation practice in South Korea, the included studies suggest the importance of integrating psychological support with functional and community-based rehabilitation. Depression screening, stress management, and psychological intervention may be important because Korean studies linked depression, stress, fear of falling, and psychological distress with QoL [32,41,43,44]. Vocational rehabilitation and employment support may also be important because paid work, economic activity, and vocational rehabilitation were associated with better QoL or HRQoL in Korean studies [26,41,43]. In addition, community transition, leisure participation, and social participation should be supported as part of long-term rehabilitation and community living [39,40].

For rehabilitation practice in the United States, the included studies suggest the importance of employment support, participation opportunities, and accessible community environments. Employment and competitive work were associated with HRQoL, social integration, and occupational outcomes [29,30,31]. Participation context, residence supports, accessibility, relationships, and personal assistance were also related to QoL and community living [35,37]. These findings suggest that social and vocational participation should remain important priorities in SCI rehabilitation and community support.

For the research community, this review highlights the need for more balanced and comparable SCI research across countries. Future studies should use clearer QoL/HRQoL definitions, standardized measures, and consistent reporting of participant characteristics such as age, injury duration, injury severity, employment status, and rehabilitation access. Future research should also examine how depression, employment, and social participation interact with health-related conditions, rehabilitation systems, and social environments across national contexts.

## 5. Conclusions

This study conducted a cross-national scoping review to identify psychosocial factors associated with QoL/HRQoL among individuals with SCI in the United States and South Korea. The findings provide a framework for understanding three key psychosocial domains that should be considered when supporting individuals with SCI across different cultural contexts: psychological adjustment, vocational participation, and social participation.

Research in South Korea more often connected QoL/HRQoL with depression, stress, fear of falling, functional independence, economic activity, vocational rehabilitation, and social attitudes. In contrast, research in the United States more often examined QoL/HRQoL in relation to employment, participation context, community living, social and job-related participation, and youth or veteran populations. These differences suggest that psychosocial adaptation to SCI should be understood not only in relation to injury-related factors but also within the sociocultural and rehabilitation contexts that shape how individuals perceive disability and adapt to life after injury.

In line with the growing emphasis on diversity and cultural context in disability and rehabilitation research, this review highlights the importance of considering cross-national perspectives when assessing QoL/HRQoL after SCI. Understanding both shared and context-specific psychosocial factors may help guide more culturally responsive rehabilitation approaches and future research on psychosocial adaptation after SCI.

## Figures and Tables

**Table 1:** Findings from the literature.

Study	Country/Domain	Design	Participants	Age/SCI Duration	QoL/HRQoL Measure	Key Findings
Jain et al. (2007) [29]	United States/Vocational domain: employment	Cross-sectional cohort study	Adults with chronic SCI (*n* = 356) recruited from VA Boston SCI Service and greater Boston area; >1 year post-SCI.	- Age: reported categorically, ≤40 to ≥70 years. - SCI duration: median 18.2 years post-injury.	A 23-item SCI-specific HRQoL questionnaire (SCI QL-23).	Employment status was independently associated with HRQoL, along with age, injury severity, and mobility.
Bergmark et al. (2008) [35]	United States/Social domain: community living/residence	Cross-sectional qualitative interview study	Adults with traumatic tetraplegia (*n* = 22) living in California and Minnesota.	- Age: mean age at injury 29.2 years (range 17–49). - SCI duration: mean 14.4 years (range 2–37).	Qualitative residence/QoL interview data.	Residence constraints and supports shaped perceived QoL; accessibility, finances, insurance, relationships, and personal assistance were central determinants.
Wilson et al. (2017) [36]	United States/Psychological domain: depression	Cross-sectional screening/secondary analysis	Persons with SCI (*n* = 210) screened in relation to depression trial.	- Age: mean 41.2 years (SD 13.8; range 18–73). - SCI duration: not summarized.	SCI-QOL Positive Affect and Well-being item bank/short form.	Spiritual well-being and depression were associated with QoL/positive affect outcomes.
Ottomanelli et al. (2013) [30]	United States/Vocational domain: employment	Prospective randomized controlled multisite trial	Veterans with SCI (*n* = 157) receiving supported employment or treatment as usual at six VA Medical Centers.	- Age: mean 48.3 years (SD 9.8). - SCI duration: mean 12.4 years post-injury (SD 11.2).	Veterans RAND 36-item Health Survey (VR-36).	Assignment to supported employment did not significantly improve HRQoL, but obtaining competitive employment was associated with better social integration, mobility, and occupation outcomes.
Kelly et al. (2012) [37]	United States/Social domain: social participation	Cross-sectional survey with correlation/regression analyses	Youth with SCI (*n* = 340) receiving care at three specialty hospitals across the United States.	- Age: mean 13.33 years (SD 3.75; range 6–18). - SCI duration: mean 5.13 years (SD 4.31).	Pediatric Quality of Life Inventory (PedsQL).	Participation context was more consistently related to QoL than frequency; different participation contexts related to emotional, social, school, and overall psychosocial QoL.
Gorzkowski et al. (2010) [38]	United States/Social/vocational/psychological domains	Cross-sectional survey/structural equation modeling	Girls with SCI (*n* = 97) receiving care at three pediatric SCI centers within one US hospital system.	- Age: mean 12.47 years (SD 3.18; range 7–17). - SCI duration: ≥1 year post-injury.	Pediatric Quality of Life Inventory (PedsQL).	Broader social participation and higher job-related participation were associated with lower depression, which in turn was associated with higher QoL.
Chapin & Holbert (2010) [31]	United States/Vocational domain: employment	Ex post facto comparative survey	Persons with SCI receiving state vocational rehabilitation services; employed/successfully rehabilitated (*n* = 36) vs. not employed/unsuccessfully rehabilitated (*n* = 31).	- Age: mean 47 years (SD 10; range 27–65) in the unsuccessful group and mean 46 years (SD 9; range 26–64) in the successful group. - SCI duration: years since injury assessed, but group summary not reported.	WHOQOL-BREF; Sense of Well-Being Inventory.	Employment at closure was associated with higher overall QoL/health and higher physical, psychological, social relationship, and environmental QoL domains.
Lee et al. (2025) [26]	South Korea/Vocational domain: employment/work	Cross-sectional latent profile analysis	Community-dwelling Korean adults with traumatic or non-traumatic SCI from InSCI survey (*n* = 711).	- Age: mean 53.36 years (SD 11.73). - SCI duration: not reported.	International SCI Quality of Life Basic Data Set/InSCI QoL sub-items.	High-QoL profile was associated with vocational rehabilitation services, paid work, less negative social attitude, and better financial status.
Moon et al. (2021) [39]	South Korea/Social domain: community transition	Program development and pre-post pilot evaluation	Four chronic SCI patients in hospital/homebound contexts receiving hospital-based transitional rehabilitation program.	- Age: 15–47 years; individual ages 47, 44, 15, and 42 years. - SCI duration: mean 736.8 days (SD 185.4).	K-WHOQOL-BREF; Korean Community Integration Questionnaire.	After the transitional rehabilitation program, K-WHOQOL-BREF and functional independence improved, supporting community-transition oriented QoL improvement.
Han et al. (2025) [40]	South Korea/Social domain: leisure/social participation	Qualitative interview study	Individuals with SCI in South Korea during COVID-19 (*n* = 15).	- Age: range 22–63 years. - SCI duration: not reported.	Qualitative exploration of QoL related to leisure, disability acceptance, autonomy, and social participation.	Leisure and social participation were described as central to QoL/well-being during COVID-19.
Shin et al. (2012) [41]	South Korea/Psychological/vocational domains	Cross-sectional observational study	Chronic SCI patients living in the Korean community (*n* = 37).	- Age: 41.5 years (SD 10.9). - SCI duration: mean 8.35 years (SD 7.0).	EQ-5D health-related quality of life.	EQ-5D and employment status differed by psychological distress group; HRQoL contributed strongly to psychological distress level.
Kim et al. (2024) [32]	South Korea/Psychological domain: depression	Prospective cross-sectional survey	SCI outpatients in South Korea (*n* = 70) with ASIA Impairment Scale C or D.	- Age: not reported. - SCI duration: mean 4.98 years (SD 5.06).	EQ-5D-5L.	QoL correlated negatively with depressive symptoms and SCI duration; regression identified fear of falling and SCI duration as significant predictors.
Kim (2014) [42]	South Korea/Psychological/social domains	Single-case intervention study	One inpatient with SCI at a hospital in Daegu; 22-year-old man with C5–6 tetraplegia (ASIA C).	- Age: 22 years. - SCI duration: not reported.	Korean WHOQOL-BREF.	Work and leisure time-use increased after intervention; depression decreased and QoL improved.
Kim (2023) [43]	South Korea/Psychological/vocational domains	Secondary analysis/descriptive correlational study	People with spinal cord disability from the 2017 Survey on Persons with Disabilities in Korea (*n* = 94).	- Age: ≥20 years. - SCI duration: not reported.	EQ-5D index.	QoL was higher with economic activity and physical activity and lower with stress and depression.
Kim & Kim (2023) [44]	South Korea/Psychological domain: depression/mental health	Randomized intervention study	Patients with SCI (*n* = 30): CBT group (*n* = 15) and control group (*n* = 15).	- Age: mean 60.47 years (SD 11.82) in the experimental group and mean 62.87 years (SD 17.02) in the control group. - SCI duration: mean 7.53 months (SD 1.80) in the experimental group and mean 7.78 months (SD 1.73) in the control group.	WHOQOL-BREF.	CBT group improved in depression, anxiety, self-efficacy, and QoL; between-group changes favored CBT.
Lee et al. (2015) [45]	South Korea/Psychological/social domains	Cross-sectional age-group comparison and hierarchical regression	Korean people with SCI (*n* = 301), divided into young adults and middle-aged adults.	- Age: 19–44 years in young adults and 45–64 years in middle-aged adults. - SCI duration: not reported.	Korean WHOQOL-BREF.	Depression influenced QoL in young adults; anxiety/depression and social factors such as income/religion influenced QoL in middle-aged adults.

## Data Availability

No new data were collected, as this study is a review study.
